# Comparison of total cold-water immersion's effects to ice massage on recovery from exercise-induced muscle damage

**DOI:** 10.1186/s40634-022-00497-5

**Published:** 2022-06-22

**Authors:** Mohammed Ali Fakhro, Fatima AlAmeen, Rim Fayad

**Affiliations:** 1 Faculty of Sport Sciences, Université Antonine, B.P. 40016, Hadat-Baabda, Lebanon; 2 Faculty of Public Health, Department of Physical Therapy, Lebanese German University, P.O Box 206, Jounieh, Lebanon

**Keywords:** Exercise-induced muscle damage, cold-water immersion, Ice massage, Muscle performance

## Abstract

**Purpose:**

The purpose of the study is to compare the effects of total cold-water immersion to ice massage on muscle damage, performance, and delayed onset of muscle soreness.

**Methods:**

Sixty participants were randomized into two groups where they completed a muscle damage protocol. Afterward, muscle damage, muscle performance, and delayed onset muscle soreness were respectively measured by serum Creatine Kinase (CK) test, one-repetition maximum (1-RM) test, countermovement jump (CMJ) test, and visual analog scale (VAS). The measurements were taken at five different timelines (Baseline, 2 H, 24 H, 48 H, and 72 H).

**Results:**

Data showed that values of all within-group measures of the dependent variables had extremely significant statistical differences (*p* < 0.001) for both intervention groups. Serum CK values peaked at 24 H for both groups. At 72 H, serum CK values dropped to baseline values in the total cold-water immersion group, while remaining high in the ice massage group. At 72 H, the values of the 1-RM test, CMJ test, and VAS approximated baseline values only in the total cold-water immersion group (*p* < 0.001).

**Conclusions:**

Total cold-water immersion (TCWI) was more effective when compared to ice massage (IM) on improving values of recovery from exercise-induced muscle damage (EIMD). Hence, this modality may be considered during athletic recovery to maximize athletic performance.

**Clinical trial registration:**

This trial was registered in ClinicalTrials.gov under the trial registration number (NCT04183816).

## Background

Exercise recovery is a crucial element in training programs that aim to maximize athletic performance and reduce injury [17]. Exercise-induced muscle damage (EIMD) is caused due to unaccustomed eccentric muscle activation, or training cycles that are performed over consecutive days [6, 27].

EIMD disrupts sarcomeres, other cytoskeletal elements, cell membranes, and impairs the excitation–contraction coupling system [25]. This disruption negatively impacts the muscle function and leads to three remarkable features (a) decrease in muscle performance; (b) delayed onset of muscle soreness (DOMS); and (c) an increase in the concentration of intramuscular enzymes in blood plasma, such as Creatine Kinase (CK) [14, 23, 25].

The EIMD process is divided into (a) mechanical and/or metabolic stress on muscle fibers and (b) the inflammatory phase [23]. Mechanical stress on the muscle fibers, after eccentric activity, leads to direct disruption of the excitation–contraction coupling system. On the other hand, metabolic stress is caused after eccentric or concentric exercises that raise the cytosolic Ca^2+^ which results in a cascade of metabolic events leading to muscle fiber degeneration [23]. Consequently, the inflammatory phase occurs to remove debris from the injured area and prepare it for regeneration [5].

Various recovery modalities including cold water immersion (CWI) and cold application have been proposed to decrease the negative effects of EIMD [4, 30]. CWI in a temperature below 15 °C can be either partial or total (except the neck and head) body immersion [1]. CWI provokes both convective cooling via blood circulation movement and conductive by direct contact of cold water with the body, resulting in a significant decrease in core temperature (Tc) [29]. The decrease in Tc leads to an increase in stroke volume through an increase in venous return and a decrease in heart rates, leading to a total increase in cardiac output. Moreover, CWI decreases muscle spasm, swelling, and fluid accumulation through a decrease in hydrostatic pressure, which results in an increase in waste removal and improving nutrient delivery which eventually speeds up the recovery [29]. Total cold-water immersion (TCWI) is more effective than partial cold-water immersion in inducing cardiovascular physiological changes [17] which is explained by conduction. The more body parts are immersed, the greater the contact with water, the better the recovery [17].

Ice massage (IM) is defined as a local massage performed by solid ice on the region of the damaged muscle [8, 11]. In contrast to CWI, IM provokes peripheral cooling through convection, leading to a modest decrease in Tc, insignificant cardiovascular alterations, and analgesic effect [17, 29].

However, there is a lack of evidence-based studies that demonstrate the rationale for these modalities to improve performance recovery [15, 29]. Many reviews have concluded that the variety in methodology regarding exercise injury, cold protocol, and performance outcomes, are responsible for the current lack of agreement in the literature [4]. Moreover, a meta-analysis conducted by Poppendieck, Faude, Wegmann, and Meyer in 2013 [18] investigated the effect of cooling on performance. The authors concluded that TCWI showed positive effects that are large enough to be relevant for competitive athletes, when used as a recovery method after sprint exercise [18].

Therefore, this study aims to compare the effects of TCWI vs IM on muscle damage, performance, and delayed onset of muscle soreness among adults, to identify the most efficient cooling modality for recovery from EIMD.

## Methods

### Design

Participants were randomly assigned to either (a) TCWI or (b) IM group, and followed up for over four days. Ethical approval was obtained from the Lebanese German University Institutional Review Board, and upon the confirmation of eligibility, each participant received an information sheet describing the study, signed voluntarily a consent form for participation, and filled a data collection form. To ensure confidentiality, participants’ data were accessible only by the principal investigators upon the need.

### Participants

Physically active Lebanese adults aged between 19 and 44 years were recruited to participate in this study. To ensure that the participants will tolerate the intensive muscle-damaging protocol, they should be engaging in mild to moderate sports activities over two to three times per week [20]. Participants with a history of serious lower limb trauma (i.e., fractures), meniscal or ligamentous tears, sensitivity to cold temperature, cardiopulmonary or inflammatory diseases, or individuals participating in strength and plyometric training exercises were all excluded.

### Procedure

The participants were recruited via WhatsApp messages sent to all the investigators’ contact lists. This message included a brief explanation of the study purpose, eligibility criteria, phone numbers of the investigators, and a request to forward this message to their contact list.

Upon eligibility, participants were familiarized with the experimental procedure. Afterward, all participants were stratified into two blocks (30 males and 30 females), and a randomization website (http://www.randomization.com) randomly allocated them equally into one of two intervention groups (TCWI or IM).

The assessment included a battery of tests including serum CK, one-repetition maximum (1-RM), countermovement jump (CMJ), and visual analog scale (VAS) respectively for baseline measurements. Testing followed the muscle damage protocol.

Firts, participants were asked to perform a knee extension exercise with the dominant lower extremity for strength testing. They started with a general warm-up of five minutes of moderate intensity jogging. This was followed by five minutes of muscle endurance exercise with a light load of 15 kg over 10 repetitions [3, 12]. Coutermovement jump testing followed strength testing after one hour. Finally, participants were asked to perform a maximal contraction of their right dominant leg (quadriceps) over four seconds, where an assessor immediately rated the level of soreness on the VAS [27].

After baseline measurements, each intervention (TCWI or IM) was conducted among the designated group. Testing was performed post muscle damage protocol at 2, 24, 48, and 72 H.

### Muscle damage protocol

The muscle damage protocol consisted of five sets of 20 drop jumps from a 60-cm high box with 2 min of rest between sets. After each drop from the box and landing on the floor, participatans were instructed to perform a maximally explosive vertical jump upward and then land on the floor. Participants were told to flex their knees to at least at 90° during all landings and to keep their hands on their hips during the jumps. Moreover, they were verbally motivated to exert maximal effort during every repetition [27].

### Recovery modalities

Participants allocated to the TCWI group completed a session of 15-min in cold water with a temperature of 12 °C [15]. Participants were seated while immersing their whole body in water except their head and neck. The temperature was continuously measured by a mercury-in-glass thermometer and was maintained at 12 °C by adding blocks of ice when needed.

Participants in the IM group were seated and subjected to a circular local massage through the use of an ice cube. Massage was applied for 15 min with no extra pressure added by the tester, for each thigh, over the region of the quadriceps muscle [17].

It is noteworthy to mention that the modalities were applied by a qualified physical therapist for all the participants.

### Data collection method

#### Serum creatine kinase

Muscle damage was tested via the serum CK blood test. This marker was chosen due to its dramatic increase in blood after EIMD, and its low cost [5]. Blood samples were taken from participants at pre-exercise, then at 2H, 24H, 48H, and 72H post-exercise for all participants in both intervention groups. The blood samples were clotted for 30 min, then centrifuged for 20 min at room temperature. The serum was then stored at -20 °C until analysis. Finally, serum CK activity was analyzed with an enzymatic method at 37 °C [27].

Muscle performance is defined as the overall capability of a muscle or muscle group [22], and is divided into (a) speed, (b) coordination, (c) dynamic balance, (d) agility, (e) flexibility, (f) muscle strength, and (g) muscle power.

#### Muscle strength

Muscle strength is defined as the maximal amount of force produced by a muscle or by a group of muscles in a specific exercise for one repetition [21]. 1-RM test, is a gold standard [23], and a reliable method for evaluating the maximal strength with test–retest measures recorded high ICC = 0.990 [12], and a confidence interval of (CI 95%) [13]. 1-RM test procedures for the measurement of muscular strength followed the recommendations of the American College of Sports Medicine [2]. The starting position of the knee extension exercise was 90-degrees of knee flexion. Weights were gradually adjusted until the subject failed to safely and correctly complete a full range of motion of extension. This load is recorded and referred to as the 1-RM [19]. A rest time of two minutes was taken between sets [2].

#### Muscle performance

Power is related to the strength and speed of movement and is defined as the work produced by a muscle per unit of time (force × distance/time) [19]. According to Markovic et al. [16], explosive muscle power is the main determinant of performance. Increase in leg power showed to affect the vertical jump height which is captured by the CMJ. CMJ begins with the knees flexed [24] and ends when the pelvis, defined by the four superior iliac spines, achieves its lowest position [20]. Therefore, participants were instructed to drop jump from a 60-cm box, then perform a maximal bipodal CMJ, then land on the floor with knees flexed to at least 90°. To ensure measurement accuracy and reliability, participants were instructed to keep their hands on their hips during the jump and to keep their legs and hips extended until contact is made (end of landing phase) [16]. CMJ was repeated three times and the highest vertical measure was recorded. ICC values showed moderate to excellent inter-session reliability for the bipodal CMJ (ICC average: 0.80, range: 0.46–0.97) according to Schwartz et al. [20] CMJ test showed excellent reliability (ICC = 0.98) and showed the highest correlation with the explosive power factor (*r* = 0.87) [16]. CMJ power values were recorded by the "My Jump" application. According to Fernández et al. [7], “My Jump” showed an excellent validity for CMJ height (*r* = 0.995, *P* < 0.001) as compared to the force platform, which is the gold standard for estimating the explosive power of the lower limbs [16]. Fernández et al. [7] stated that there was almost perfect agreement between the force platform and “My Jump” for the CMJ height (ICC = 0.997, *P* < 0.001; Bland–Altman bias = 1.1 ± 0.5 cm, *P* < 0.001).

#### Muscle soreness

DOMS is defined as a delayed feeling of soreness (about 24H after the exercise) accompanied by muscle stiffness, aching soreness, and/or muscular tenderness [14]. Muscle soreness peaks within 72H and slowly resolve within 5 to 7 days [14]. DOMS was assessed by the VAS of 10 degrees, ranging from “no soreness” (0) to “severe soreness” [9] [27]. VAS showed excellent reliability in the assessment of DOMS, the ICC range was 0.98–0.99 for VAS [10].

### Satistical analysis

Data were analyzed using the Statistical Package for the Social Sciences (SPSS) version 21.0 for windows. Descriptive statistics (mean, standard deviation) were calculated for participants’ age and the main key variables of the study. The Shapiro–Wilk test was used to check the normality of the data distribution. A probability (p) value of > 0.05 indicates that there is no significant difference between groups.

Regarding data analysis, a two-way (group x time) mixed factor analysis of variance (ANOVA) test was used to compare the mean differences between groups, TCWI and IM, for each dependent variable (CK, 1-RM, CMJ, and DOMS) at different timelines (Baseline, 2 H, 24 H, 48 H, and 72 H). Moreover, the T-test was used to indicate the significance of the difference between variables.

## Results

The mean of age of the 60 participants was 24 ± 2.9 years. Mean values of each dependent variables (serum CK, 1-RM, CMJ, and VAS) were compared at five different timelines. Recorded data showed extremely significant within-group differences for all of CK, muscle strength, muscle power, and DOMS (*p* = 0.000) (Table 1).

**Table 1:** Computation of within and between groups statistical significance of dependent variables at different timelines

Measure	Timeline	TCWI		IM		Significance
		**Mean**	**Standard deviation**	**Mean**	**Standard deviation**	**Between-group (p-value)**
CK (U/L)	Baseline	103.53	13.42	107.43	14.58	0.286
	2 H	219.57	58.82	236.73	75.73	0.331
	24 H	275.43	64.27	289.63	52.88	0.354
	48 H	174	25.97	235.3	44.002	< 0.001^2^
	72 H	109.7	12.94	190.77	33.09	< 0.001^2^
	**Within-group (*p*-value)**	< 0.001^2^		< 0.001^2^		
1-RM (Kg)	Baseline	22.7	8.24	22.51	8.16	0.931
	2 H	16.66	8.31	17.53	8.04	0.683
	24 H	18.71	8.11	13.86	7.49	0.0191
	48 H	20.8	8.81	10.78	7.05	< 0.001^2^
	72 H	22.65	8.23	15.35	6.94	< 0.001^2^
	**Within-group (*p*-value)**	< 0.001^2^		< 0.001^2^		
CMJ (Cm)	Baseline	23.65	6.95	23.65	6.52	0.871
	2 H	15.4	4.6	15.3	3.26	0.923
	24 H	18.16	4.9	11.58	3.2	< 0.001^2^
	48 H	21.13	21.13	7.98	3.13	< 0.001^2^
	72 H	23.63	4.88	13.61	2.68	< 0.001^2^
	**Within-group (*p*-value)**	< 0.001^2^		< 0.001^2^		
DOMS		0	0	0	0	0.431
	2 H	2.7	0.98	3.27	0.78	0.0171
	24 H	7.23	0.93	7	0.87	0.321
	48 H	4	1.01	8.27	0.69	< 0.001^2^
	72 H	0.37	0.61	4.87	1.25	< 0.001^2^
	**Within-group (*p*-value)**	< 0.001^2^		< 0.001^2^		

*TCWI* total cold-water immersion, *IM* Ice massage, *CK* Creatine Kinase, *1-RM* one-repetition maximum, *CMJ* Countermovement jump, *DOMS* delayed onset muscle soreness, *P*-value: probability value, 1 Significant, 2 Extremely significant

**Fig. 1:**
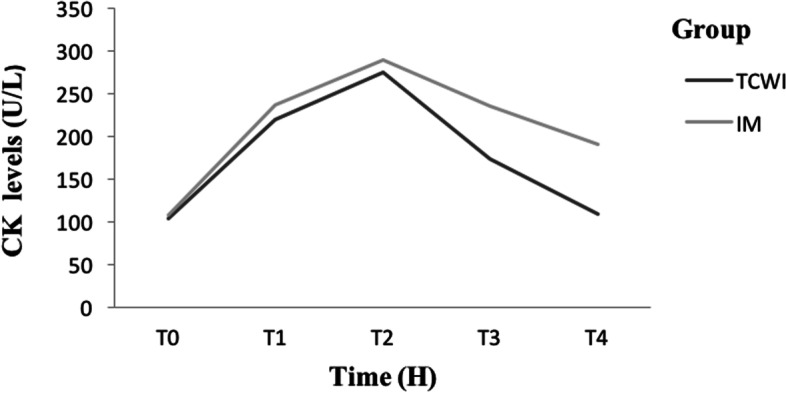
Variation of creatine kinase's activity between groups at different timelines

The CK between-groups mean values at baseline were not statistically significant (*p* = 0.289) (Table 1). At 24 H serum CK recorded a peak for both groups with a mean value of 275 ± 64 U/L for TCWI and 289 ± 52 U/L for IM, and started to drop at 48 H and onward where it was statistically significant lower in the TCWI group (Table 1; Fig. 1).

**Fig. 2:**
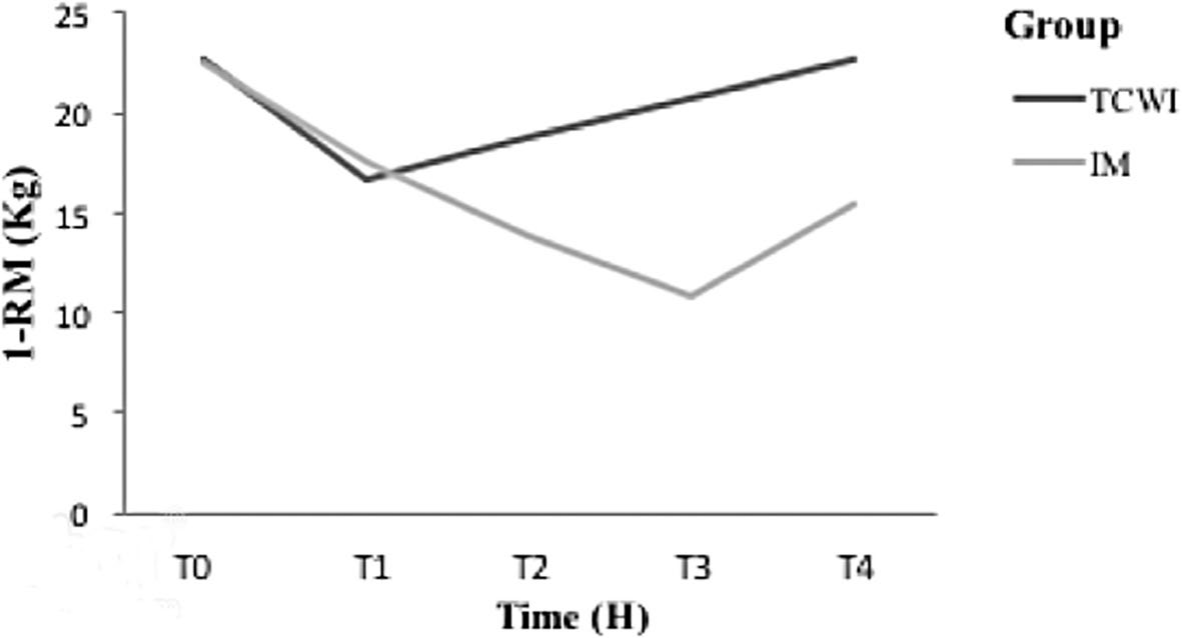
Variation of one-repetition maximum's values between groups at different timelines

1-RM values dropped at 2 H for both groups as compared to baseline values, 22 ± 8 kg for TCWI, and 22 ± 8 kg for IM (Table 1). However, at 48 H, the strength value increased only for the TWCI group while it continued to drop for IM significantly. Finally, at 72 H strength values started to increase again in the IM group, while returning approximately to baseline values in the TWCI group (*p* < 0.001) (Table 1; Fig. 2).

**Fig. 3:**
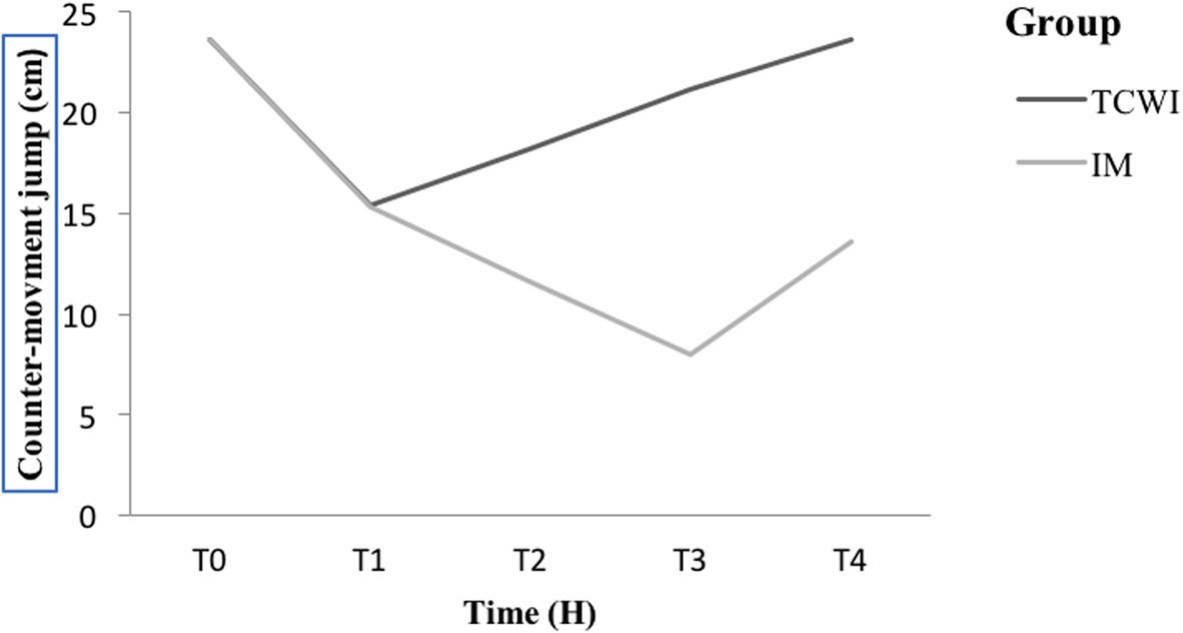
Variation of countermovement jump's height between groups at different timelines

Regarding CMJ, both groups showed a non-significant statistical decrease in the values recorded at 2 H, as compared to baseline values (Table 1). However, in the TCWI group, values started to increase again at 24 H to reach approximately baseline values at 72 H (*p* < 0.001). On the other hand, values in the IM group continued to decrease at 24 H and 48 H, to rise again at 72 H (*p* < 0.001) (Table 1; Fig. 3).

**Fig. 4:**
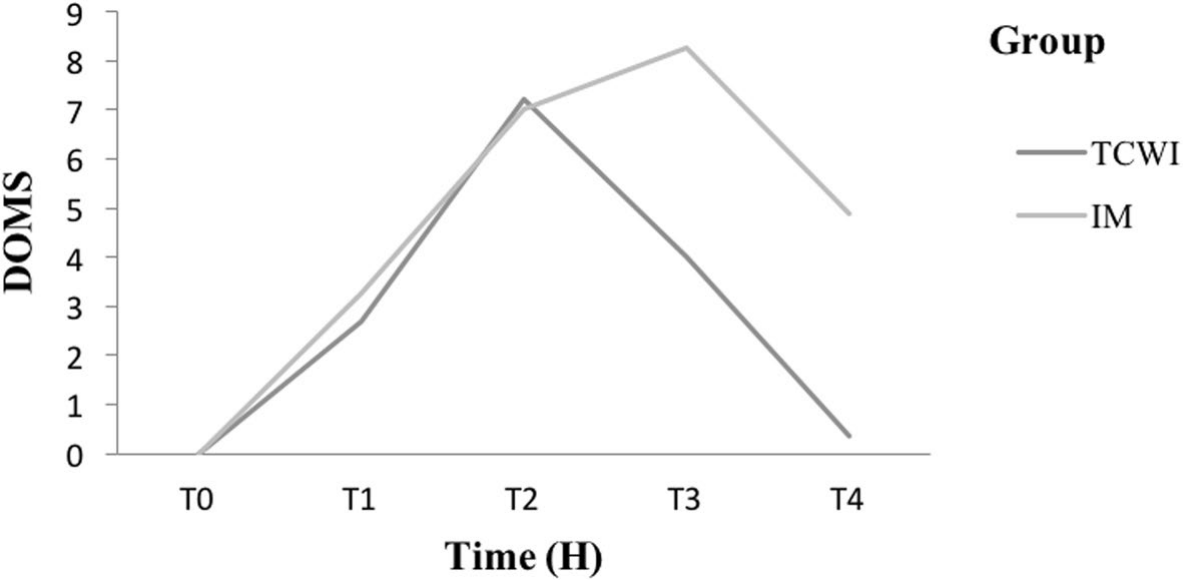
Variation of delayed onset of muscle soreness values between groups at different timelines

VAS values continued to increase to reach a peak for the TCWI group (7.23 ± 0.93) at 24 H, to return to the baseline value at 72 H. However, in the IM group, the VAS mean values continued to increase until 48 H, and didn’t ultimately return to baseline value at 72 H (*p* < 0.001) (Table 1; Fig. 4).

## Discussion

Both groups showed an increase in the serum CK levels, which peaked after 24 H and then dropped again to return to baseline values in the TCWI group while remaining higher at all times in the IM groups. This indicates that TCWI intervention had a better effect on muscle damage than IM. This is in line with other studies that showed that TCWI reduces the concentration of serum CK after 24 H [1, 26]. Moreover, Howatson et al. [8] compared the effect of IM to placebo on muscle damage. Findings showed that CK concentration in the IM group dropped but without returning to pre-exercise values, while that of the placebo group kept increasing until 96 H. This indicates that IM has a better effect than placebo, however, full recovery was not achieved before 96 H. Therefore, both IM and TCWI affect recovery from muscle damage, but TCWI leads to full recovery in a shorter time.

Muscle strength declined in both groups 2 H after the muscle damage protocol. However, strength was recovered and continued to improve after 24 H in the TCWI group while it was declining in the IM group to recover again at 72 H. This shows that muscle recovery is faster among the TCWI group. Similarly, many studies highlighted the benefit of TCWI on muscle strength recovery [9, 28].

Two hours after the damaging protocol, muscle power dropped among both groups. However, power was recovered and continued to improve at 24 H in the TCWI group, and returned to normal values after 72 H. Vieira et al. [27] stated that the muscular pre-stretch activation before the propulsive phase of CMJ combines several neuromuscular mechanisms, including more time available to increase force, storage of elastic energy, potentiation of contractile machinery, and activation of proprioceptive reflexes. It was shown that TCWI enhanced the above-mentioned neuromuscular mechanisms, and led to a better recovery of muscle power performance.

Muscle soreness peaked at 24 H in both groups. Afterward, soreness dropped among participants in the TCWI group to return to normal levels after 72 H, while kept fluctuating among those in the IM group. These findings matches those of similar studies [27]. However, Vieira et al. [27] applied TCWI intervention several times (baseline, 4 H, 8 H, and 24 H) and used relatively longer durations (2 bouts of 15 min), which demonstrated better impact on the recovery from DOMS.

One of the study’s limitations was that one of the indicators of EIMD, inflammation, was not included among the variables tested. Furthermore, data collected were from the adults (19 to 44 years) subgroup of the population. This fact might limit the generalizability of the findings to the remaining age groups.

## Conclusion

TCWI showed superior benefits compared to IM regarding muscle damage, muscle power, muscle strength, and DOMS. All key variables returned to baseline values in the TCWI group at 72 H after the muscle-damaging protocol. Therefore, the TCWI modality can be integrated into the training programs to accelerate recovery from EIMD.

## Institutional review board statement

The study protocol was approved by the Institutional Review Board (EC Ref No.: 3EC/2019) of the Lebanese German University in Sahel Alma, Jounieh, Lebanon.

## CONSORT 2010 statement

The authors have read the CONSORT 2010 statement, and the manuscript was prepared and revised according to the CONSORT 2010 statement.

## Data Availability

The datasets used and/or analyzed during the current study are available from the corresponding author on reasonable request.

## Abbreviations

CK: Creatine Kinase
1-RM: One-repetition maximum
CMJ: Countermovement jump
VAS: Visual analog scale
EIMD: Exercise-induced muscle damage
CWI: Cold-water immersion
TCWI: Total cold-water immersion
IM: Ice massage

## References

[1] Abaïdia AE, Lamblin J, Delecroix B (2016). Recovery from exercise-induced muscle damage: cold water immersion versus whole body cryotherapy. Int J Sports Physio and Performance.

[2] American College of Sports Medicine. ACSM’s guidelines for exercise testing and prescription. 10^th^ ed. Philadelphia, PA: Wolters Kluwer. 2018

[3] Barnett A (2006). Using recovery modalities between training sessions in elite athletes: does it help?. Sports Med.

[4] Chen CH, Chen TC, Jan MH, Lin JJ (2014). Acute Effects of Static Active or Dynamic Active Stretching on Eccentric-Exercise-Induced Hamstring Muscle Damage. Int J Sports Phys and Performance.

[5] Clarkson PM, Hubal MJ (2002). Exercise-induced muscle damage in humans. Am J Phys Med Rehabil.

[6] Davies RC. The Effects of Exercise-Induced Muscle Damage on the Human Response to Dynamic Exercise. Thesis for the degree of Doctor of Philosophy in Sport and Health Sciences. University of Exeter. 2010

[7] Fernández CB, Glaister M, Lockey RA (2014). The validity and reliability of an iPhone app for measuring vertical jump performance. J Sports Sciences.

[8] Howatson G, Gaze D, Van Someren KA (2005). The efficacy of ice massage in the treatment of exercise-induced muscle damage. Scand J Med Sci Sports.

[9] King M, Duffield R (2009). The effects of recovery interventions on consecutive days of intermittent sprint exercise. J Strength Cond Res.

[10] Lau WY, Muthalib M, Nosaka K (2013). Visual analog scale and pressure soreness threshold for delayed onset muscle soreness assessment. J Musculoskeletal Soreness.

[11] Lea J (2014). The effects of knee-high compression garments and IM on exercise performance and recovery.

[12] LeBrasseur NK, Bhasin S, Miciek R, Storer TW (2008). Tests of Muscle Strength and Physical Function: Reliability and Discrimination of Performance in Younger and Older Men and Older Men with Mobility Limitations. J Am Geriatr Soc.

[13] Levinger I, Goodman C, Hare DL, Jerums G, Toia D, Selig S (2009). The reliability of the 1RM strength test for untrained middle-aged individuals. J Sci Med Sport.

[14] Lewis PB, RubyD, Bush-Joseph C. Muscle soreness, and delayed-onset muscle soreness. Clin Sports Med. 2012;31(2):255–262. 10.1016/j.csm.2011.09.009 10.1016/j.csm.2011.09.009 22341015

[15] Machado AF, Almeida AC, Micheletti JK (2016). Dosages of cold-water immersion post-exercise on functional and clinical responses: a randomized controlled trial. Scand J Med Sci Sports.

[16] Markovic G, Dizdar D, Jukic I, Cardinale M (2004). Reliability and factorial validity of squat and countermovement jump tests. J Strength and Conditioning Research.

[17] Murray A, Cardinale M (2015). Cold applications for recovery in adolescent athletes: a systematic review and meta-analysis. Extrem Physiol.

[18] Poppendieck W, Faude O, Wegmann M, Meyer T (2013). Cooling and Performance Recovery of Trained Athletes: A Meta-Analytical Review. Int J Sports Physiol Perform.

[19] Puthoff ML. The relationship between impairments in muscle performance functional limitations, and disability in older adults. PhD thesis, University of Iowa. 2006; 10.17077/etd.q4ja0qok.le

[20] Schwartz C, Forthomme B, Paulus J (2017). Reliability of unipodal and bipodal countermovement jump landings in a recreational male population. Eur J Sport Sci.

[21] Stoppani, J. Encyclopedia of Muscle & Strength. Human Kinetics, Champaign, III. 2006

[22] Swain DP (2014). ACSM’s Resource Manual for Guidelines for Exercise Testing and Prescription.

[23] Tee JC, Bosch AN, Lambert MI. Metabolic consequences of exercise-induced muscle damage. Sports Med. 2007;37(10):827–836. 0112–1642/07/0010–0827 10.2165/00007256-200737100-00001 17887809

[24] Thomas K, French D, Hayes PR (2009). The effect of two plyometric training techniques on muscular power and agility in youth soccer players. J Strength and Cond Research.

[25] Tiidus PM. Skeletal Muscle Damage and Repair. Waterloo, Ontario, Canada: Human Kinetics. 2008

[26] Vaile J, Halson S, Gill N, Dawson B (2008). Effect of hydrotherapy on the signs and symptoms of delayed onset muscle soreness. Eur J Appl Physio.

[27] Vieira A, Siqueira AF, Ferreira-Junior JB (2016). The effect of water temperature during cold-water immersion on recovery from exercise-induced muscle damage. Int J Sports Med.

[28] White GE, Rhind SG, Wells (2014) The effect of various cold-water immersion protocols on exercise-induced inflammatory response and functional recovery from high-intensity sprint exercise. Eur J Appl Physiol 114(11):2353–2367. 10.1007/s00421-014-2954-2 10.1007/s00421-014-2954-2 25074283

[29] White GE, Wells GD (2013). Cold-water immersion and other forms of cryotherapy: physiological changes potentially affecting recovery from high-intensity exercise. Extreme Phys & Med.

[30] Demirhan B, Yaman M, Cengiz A, Saritas N, Günay M (2015) Comparison of Ice Massage versus Cold-Water Immersion on Muscle Damage and DOMS Levels of Elite Wrestlers. The Anthropologist 19(1):123-129. 10.1080/09720073.2015.11891646

