# Correction: Comparison of total cold-water immersion’s effects to ice massage on recovery from exercise-induced muscle damage

**DOI:** 10.1186/s40634-022-00532-5

**Published:** 2022-09-16

**Authors:** Mohammed Ali Fakhro, Fatima AlAmeen, Rim Fayad

**Affiliations:** 1 Faculty of Sport Sciences, Université Antonine, B.P. 40016, Hadat-Baabda, Lebanon; 2 Faculty of Public Health, Department of Physical Therapy, Lebanese German University, P.O Box 206, Jounieh, Lebanon

**Correction: J Exp Ortop 9, 59 (2022)**

10.1186/s40634-022-00497-5

Following publication of the original article [1], the authors identified an error in reference 30. The reference 30 should be:

Demirhan B, Yaman M, Cengiz A, Saritas N & Günay M (2015) Comparison of Ice Massage versus Cold-Water Immersion on Muscle Damage and DOMS Levels of Elite Wrestlers, The Anthropologist, 19:1, 123-129, DOI: 10.1080/09720073.2015.11891646

Instead of:

Yaman M, Nazmi S & Cengiz A (2015) Comparison of Ice Massage versus Cold-Water Immersion on Muscle Damage and DOMS Levels of Elite Wrestlers. Anthropologist. DOI:10.1080/09720073.2015.11891646

The original article has been corrected.
